# Ethnic variations in incidence of asthma episodes in England & Wales:national study of 502,482 patients in primary care

**DOI:** 10.1186/1465-9921-6-120

**Published:** 2005-10-21

**Authors:** Gopalakrishnan Netuveli, Brian Hurwitz, Aziz Sheikh

**Affiliations:** 1 Department of Primary Care & Social Medicine, Imperial College London, London W6 8RP, UK; 2 School of Humanities, King's College London, Strand, London WC2R 2LS, UK; 3 Division of Community Health Sciences: GP Section, University of Edinburgh, 20, West Richmond Street, Edinburgh EH8 9DX, UK

## Abstract

**Background:**

Recent studies have demonstrated marked international variations in the prevalence of asthma, but less is known about ethnic variations in asthma epidemiology within individual countries and in particular the impact of migration on risk of developing asthma. Recent within country comparisons have however revealed that despite originating from areas of the world with a low risk for developing asthma, South Asian and Afro-Caribbean people in the UK are significantly (3× and 2× respectively) more likely to be admitted to hospital for asthma related problems than Whites.

**Methods:**

Using data from the Fourth National Study of Morbidity Statistics in General Practice, a one-percent broadly representative prospective cohort study of consultations in general practice, we investigated ethnic variations in incident asthma consultations (defined as new or first consultations), and compared consultation rates between those born inside and outside the UK (migrant status). Logistic regression models were used to examine the combined effects of ethnicity and migration on asthma incident consultations.

**Results:**

Results showed significantly lower new/first asthma consultation rates for Whites than for each of the ethnic minority groups studied (mean age-adjusted consultation rates per 1000 patient-years: Whites 26.4 (95%CI 26.4, 26.4); South Asians 30.4 (95%CI 30.3, 30.5); Afro-Caribbeans 35.1 (95%CI 34.9, 35.3); and Others 27.8 (27.7, 28.0). Within each of these ethnic groups, those born outside of the UK showed consistently lower rates of incident asthma consultations. Modelling the combined effects of ethnic and migrant status revealed that UK-born South Asians and Afro-Caribbeans experienced comparable risks for incident GP consultations for asthma to UK-born Whites. Non-UK born Whites however experienced reduced risks (adjusted OR 0.82, 95%CI 0.69, 0.97) whilst non-UK born South Asians experienced increased risks (adjusted OR 1.33, 95%CI 1.04, 1.70) compared to UK-born Whites.

**Conclusion:**

These findings strongly suggest that ethnicity and migration have significant and independent effects on asthma incidence. The known poorer asthma outcomes in UK South Asians and Afro-Caribbeans may in part be explained by the offspring of migrants experiencing an increased risk of developing asthma when compared to UK-born Whites. This is the first study to find heterogeneity for incident asthma consultations in Whites by migrant status.

## Background

The International Study of Asthma and Allergies in Childhood (ISAAC) and European Community and Respiratory Health Survey (ECRHS) have revealed marked international variations in the prevalence of asthma, with populations living in economically-developed countries experiencing the highest prevalence rates [1,2]. The precise reasons underpinning these variations are poorly understood, but point in particular to the possible importance of differing exposures to environmental risk factors.

In contrast to marked international variations in the prevalence of asthma, a recent systematic review and meta-analysis of epidemiological studies *within *the UK has found that despite originating from low risk areas internationally, South Asians and Afro-Caribbeans experience significantly poorer asthma outcomes than do Whites [3]. Possible reasons for these poorer outcomes could include differences in asthma incidence, severity, management and/or health seeking behaviour between ethnic groups [4-8].

In this study, we investigated possible ethnic variations in incidence of asthma episodes and in addition explored the impact of migration on risk of developing asthma. We hypothesised that the incidence of asthma episodes would vary between ethnic groups with those born in low risk regions experiencing a lower incidence than those born in the UK (a very high risk region).

Whilst some of these results have previously been presented in summary format in our systematic review and meta-analysis of ethnic variations in the epidemiology and outcomes of asthma in UK minority ethnic groups [3], the background, rationale, methods and detailed results have never previously been reported in the peer-review literature.

## Methods

### Study sample

Our study sample consisted of patients included in the Fourth National Study of Morbidity Statistics in General Practice (MSGP4), a year long prospective cohort study during the period September 1991 – August 1992 of more than half a million patients registered with 60 general practices in England and Wales [9]. The study sample was a broadly representative one-percent sample of the general population of England and Wales.

### Definitions and measures

Ethnicity was coded using 1991 census categories [10]. Because of small numbers in some ethnic groups, we re-categorised these data into four broader ethnic groups: 'Whites', 'South Asians' 'Afro-Caribbeans' and 'Others'. In doing so, we combined Indians, Pakistanis and Bangladeshis into 'South Asians', while assigning Chinese to the 'Others' group. Immigrants were defined as all those born outside of the UK irrespective of nationality or ethnicity.

A 'consultation' was defined as a face-to-face encounter between a patient and a member of the practice clinical staff leading to at least one Read code diagnosis. Read codes were mapped onto the ninth revision of the International Classification of Diseases (ICD-9) [11]. If the diagnostic code was for asthma (ICD-9 493), we defined this as being an asthma consultation. We marked asthma consultations as 'first' if the patient had never before consulted for asthma during their life time and 'new' if the patient had previously consulted for asthma, but not in the last 28 days. All those consultations marked 'first' by definition mark 'new' episode consultations for asthma and hence, by considering 'first' and 'new' consultations together we were able to examine the incidence of asthma episodes. However, not all registered patients consulted during the MSGP4 study period and some patients consulted more than once. Furthermore, due to births, deaths and patient mobility, the denominator was not constant over the study period. We therefore calculated the 'patient years at risk' by dividing the number of days for which patients were registered with a practice during the 12-months study period by the number of days in the year (366 in 1991–92).

As general explanatory variables, we used age, sex, Registrar General's social class, dichotomised as non-manual (Classes I, II, IIINM) and manual (Classes IIIM, IV, V), urban, and current smoking status which was assessed by a single question ('smoked in the previous week').

### Statistical analysis

The incidence rates for asthma episodes were calculated using episodes defined as new or first consultations per person years at risk. Ethnic minority groups in the UK are known to be significantly younger than the White majority population [12]; we therefore adjusted for age by standardising against the age structure of the total sample. As a sensitivity analysis, we also examined age-specific rates.

We used logistic regression modelling to examine for the effects of ethnicity and migrant status on asthma incidence. As explanatory variables, we used a categorical variable for ethnicity (Whites, South Asians, Afro-Caribbeans and Others) and a binary variable to represent whether subjects were born inside/outside the UK. Three models, containing ethnicity alone, immigrant status alone and ethnicity and immigrant status together, were fitted. The dependent variable in our regression models was the presence or absence of a new or first consultation for asthma episodes. The possibility of confounding and/or effect modification was assessed using the following covariates: age, sex, social class, house ownership, urban area and current smoking status.

We undertook a sensitivity analysis to test the hypothesis that data on ethnicity were missing more in non-White ethnic groups (selection bias) using the Heckman procedure [13]. Briefly, we created a binary variable denoting absence of ethnicity information and used age, sex, manual social class and smoking status to model this variable. This selection model was fitted together with the substantive model using new/first asthma episode consultations as the dependant variable and ethnicity and migration status as predictor variables. The parameter of interest was the correlation between error terms in the substantive model and the selection model which might indicate significant selection bias in the sample. All analyses were undertaken using Stata Version 7 [14].

## Results

Information on ethnicity was available for 82.7% (n = 415,528) of subjects, those with missing data experiencing significantly lower new/first asthma episode consultation rates than the overall sample (Table 1). Of these, 2.4% (n = 9,981) belonged to non-White ethnic minority groups.

**Table 1:** Sample description and new/first consultations for asthma episodes (incidence) by ethnicity and place of birth

**Ethnicity**	**Country of birth**	**Total number of patients (%)**	**Mean age, years (sd)**	**Asthma**			
				
				**Total consultations/1000 patient years**	**New/first Consultations/1000 patient years**		
				
				**Unadjusted**	**Unadjusted**	**Age-adjusted**	
				
				**Mean**	**Mean**	**Mean**	**95%CI**
Whites	UK	393,300 (78.3)	36 (23)	35.7	27.1	26.5	26.2 to 26.8
	Non-UK	12,247 (2.4)	43 (21)	29.3	19.8	24.9	23.3 to 26.4
	Total	405,547 (80.7)	37 (23)	35.5	26.9	26.4	26.4 to 26.4
South Asians	UK	2,187 (0.4)	12 (11)	54.5	46.3	34.7	26.0 to 43.5
	Non-UK	3,501 (0.7)	38 (16)	27.6	24.6	23.7	20.2 to 27.3
	Total	5,688 (1.1)	28 (19)	37.9	32.9	30.4	30.3 to 30.5
Afro-Caribbeans	UK	1,428 (0.3)	19 (15)	61.2	52.3	39.2	31.5 to 46.8
	Non-UK	1,080 (0.2)	40 (16)	28.0	22.0	20.4	14.2 to 26.5
	Total	2,508 (0.5)	28 (19)	47.0	39.3	35.1	34.9 to 35.3
Others	UK	932 (0.2)	13 (13)	73.0	38.2	30.9	18.6 to 43.1
	Non-UK	853 (0.2)	32 (16)	27.8	27.8	30.3	23.2 to 37.4
	Total	1,785 (0.4)	22 (18)	51.9	33.4	27.8	27.7 to 28.0
Missing		86,954 (17.3)	36 (21)	16.4	11.8	13.4	12.9 to 13.8
**Total**		**502,482 (100)**	**36 (23)**	**35.7**	**23.7**	**24.8**	**24.3 to 25.2**

Age-standardised analysis revealed that new/first asthma episode consultation rate for Whites was significantly lower than for South Asians, Afro-Caribbeans and Others. When these ethnic groups were subdivided into those born inside and outside of the UK, those born outside of the UK were found to experience significantly lower rates of new/first episode consultations for asthma (Table 1).

Unadjusted models for ethnicity showed that non-White ethnic minorities had greater risk of experiencing a new/first asthma episode consultation than did Whites; however after adjusting for important confounders only South Asians were found to be at increased risk of new/first asthma episode consultations (OR 1.33, 95%CI 1.06, 1.67) (Table 2).

**Table 2:** Logistic regression models for relationship between ethnicity, immigrant status and risk of new/first consultation for asthma episodes

	**Odds ratios for new/first consultation for asthma**			
	
	**Unadjusted**		Adjusted*	
	
	**OR**	**95% CI**	**OR**	**95% CI**
***Ethnicity***				
Whites	1		1	
South Asians	1.21	1.04 to 1.41	1.33	1.06 to 1.67
Afro-Caribbeans	1.44	1.17 to 1.78	1.04	0.73 to 1.48
Others	1.64	1.30 to 2.07	1.12	0.68 to 1.83
***Immigrant status***				
Born outside UK	0.75	0.67 to 0.83	0.93	0.82 to 1.06
***Ethnicity and immigrant status***				
White, UK-born	1		1	
White, non-UK born	0.71	0.62 to 0.81	0.82	0.69 to 0.97
South Asian, UK-born	1.71	1.39 to 2.10	1.48	0.81 to 2.70
South Asian, non-UK born	0.89	0.71 to 1.11	1.33	1.04 to 1.70
Afro-Caribbean, UK-born	1.94	1.52 to 2.46	1.30	0.79 to 2.13
Afro-Caribbean, non-UK born	0.78	0.51 to 1.19	0.96	0.57 to 1.60
Other, UK-born	2.27	1.72 to 2.99	1.47	0.65 to 3.37
Other, non-UK born	0.95	0.61 to 1.46	0.80	0.40 to 1.61

*Adjusted for age, sex, social class, house ownership, urban and smoking status

Unadjusted models for migrant status showed that for each of the ethnic groups those born outside the UK showed reduced risk of incident asthma episodes in primary care compared with those born in the UK, and this relationship was retained after adjusting for age differences except in the Others group (Table 1), but was no longer found in any of the groups when adjusted for differences in age, sex, social class, house ownership, urban rural location and smoking status (OR 0.93, 95%CI 0.82, 1.06) (see Table 2).

When ethnicity and migrant status were considered together, there were no significant differences in odds ratios for incident asthma episodes between UK-born Whites, UK-born South Asians and (all) Afro-Caribbeans (Table 2). However, non-UK born Whites had a reduced risk of new/first asthma episode consultation (OR 0.82, 95%CI 0.69, 0.97) compared with UK born Whites, whereas non-UK born South Asians experienced an increased risk (OR 1.33, 95%CI 1.04, 1.70) compared with UK born Whites.

Sensitivity analysis using Heckman sample selection modelling revealed no evidence of sample selection bias (p = 0.40).

## Discussion

This large national study has found that UK ethnic minority groups experience significantly higher age-adjusted new/first episode consultation rates for asthma than the White majority population, this suggesting significant variation in asthma incidence between ethnic groups. Taken together with our finding of significantly lower age-adjusted rates of incident episodes in those (including Whites) born outside the UK and the known patterns of migration to the UK, these findings reinforce the likely importance of early life environmental exposures in influencing risk of developing, and subsequent outcomes for, one of the commonest chronic disorders worldwide.

Table 1 reveals that the higher incident consultation rates in minority ethnic groups is driven by those born inside the UK; being born outside of the UK is associated with lower asthma consultation rates. There are three main explanations which could account for these findings: differing demographies of the populations being compared, a genuinely increased risk resulting from social (environmental) exposures in those born in the UK and differences in health seeking behaviour between groups. Comparison of the unadjusted and age-adjusted rates in Table 1 shows that population age structure is an important consideration, but that not all of the differences are accounted for by age. Turning to Table 2, the unadjusted analyses reveal that ethncitiy and migration status are both important, with UK born subjects behaving very differently from non-UK born subjects except for the heterogeneous Other group. These findings are unlikely to be the result simply of differing age or other demographic factors modelled in the analysis, as the adjusted point estimates demonstrate that these factors do not explain the entire effect.

Ethnicity has been established as an important variable in asthma epidemiology; but ethnic identity is not homogenous (often due to inadequate definitions) [15]. We have shown that migrant status in three of the main UK ethnic groups – Whites, South Asians and Afro-Caribbeans – contributes significantly to the heterogeneity of incident consultation for asthma in primary care, a finding that strengthens the possibility that there is both a spatial and a temporal effect on asthma incidence in ethnic groups.

Importantly, our findings contrast with those of the European Community Respiratory Health Survey study, which found no significant differences in utilisation of health services by migrants, and no pattern in the prevalence of asthma symptoms after taking account of asthma prevalence in the regions of origin and in the host country [16].

The limitations of our study need to be appreciated. No data on ethnicity were recorded on 17.3% of subjects consulting for asthma in the MSGP4 who were therefore excluded from our analysis. The excluded subjects had lower rates of new/first episodes of asthma than those on whom ethnicity data were available (Table 1), which could represent an important source of bias. The results of the sensitivity analysis, however, make it unlikely that ethnicity information was systematically more commonly withheld by non-White *and/or *non-UK born subjects (but caution is in order because ethnic minorities constituted 2.4% of the population sample in our study compared to 6% reported in the 1991 census) [17]. Also of potential relevance is that using MSGP4 data to estimate asthma episode incidence involves use of a surrogate, as new consultations for asthma refer to practice episodes rather than patient episodes; therefore some patients coded as having a new/first episode of asthma may already have had asthma diagnosed in a previous practice. It is however important to appreciate that in creating MSGP4 data, a very diligent edit process is used, enabling MSGP4 authors to maintain that "all first and new episode rates indicate incidence of a condition or group of conditions" [9]. Another potential limitation is that such incidence data relate to episodes rather than to persons, but such episode incidence data are acceptable for the purposes of comparing different groups based on ethnicity and migrant status. It s also important to acknowledge that in using MSGP4 consultations to elicit asthma episodes our results could be confounded by behavioural factors that influence consultations. In defence of our study it should be pointed out that we used only consultations for asthma as diagnosed by the clinician and within each ethnic group the impact of migrant status remains similar (and this was also true for the total number of consultations for asthma (data not shown)). One should also take into account limitations in the generalisability of the MSGP4 practice sample: participating practices in the survey were not entirely representative of the distribution of practices in England and Wales in 1991. Despite this, there is good agreement on asthma consultation rates between MSGP4 and asthma diagnosis rates in the General Practice Research Database [18]. An important limitation in our study is the lack of information on length of UK residence, as immigrant asthma prevalence is reported to become closer to that in the host country population with increasing length of residence [19-21].

The strengths of this dataset are however substantial and include its prospective cohort design, the broadly representative large sample size, and the availability of data on a range of potential confounders and effect modifiers in the relationship between ethnicity, migration and asthma consultations. For example, ethnic minorities in the UK are on the whole significantly younger than the White majority population, but we were able to standardise for this when calculating new/first asthma episode consultation rates thus allowing meaningful comparisons to be made across ethnic groups.

Results which echo our findings have been reported in other countries. It has been shown that those born outside of western countries are at reduced risk of asthma-related hospital admissions [22,23]. In Germany, greater cultural adaptation has been shown to be associated with increased prevalence of asthma in Turkish children [24], these findings also pointing to the importance of ethnic and migration related factors. There is some evidence that such factors might be lifestyle related; while South Asian children born in Africa, who have a more westernised lifestyle, had asthma prevalence similar to South Asian children born in the UK [25], South Asian children born in South Asia had a lower prevalence [26].

Our study found significant differences based on migration status. It is possible that migrants bring with them the same pattern of morbidity and health service utilisation as they experience in their country of origin. However, interpreting this finding is difficult since there are several possible types of explanation, including genetic, immunity from early exposures in native country, lack of exposure to factors in the host country, and selection through the healthy migrant effect [27-29].

## Conclusion

This large national study suggests that poorer asthma outcomes may in part be explained by the offspring of South Asian and Afro-Caribbean migrants to the UK experiencing an increased risk of developing asthma compared to UK-born Whites.

## Abbreviations

ECRHS – European Community and Respiratory Health Survey

ISAAC – International Study of Asthma and Allergies in Childhood

MSGP4 – 4th Morbidity Survey in General Practice

UK – United Kingdom

## Conflict of interest

The author(s) declare that they have no competing interests.

## Contributorship

GN and AS conceived this study and together with BH formulated the study protocol. GN performed data analysis. All three authors were involved in interpreting results and writing the paper. GN is guarantor.

## Funding

GN was employed on a grant from Asthma UK (01/018) when he undertook this work.

## Ethics approval

Not required
